# The Promise of a Golden Era for Exploring the Frontiers of Aging, Metabolism and Redox Biology

**DOI:** 10.3389/fragi.2020.610406

**Published:** 2020-11-16

**Authors:** Jianhua Zhang

**Affiliations:** Department of Pathology, Center for Free Radical Biology, University of Alabama at Birmingham, Birmingham, AL, United States

**Keywords:** autophgy, mitophagy, diet, exercise, omics, bioenergetics, oxidative stress, comparative biology

From ancient times to the modern day extending longevity or even finding the elixir for eternal life has been a motivating quest for many civilizations. There are no shortage of Hollywood films and TV series that feature long-lived creatures: some heroes and others villains. Many of the ancient Greeks have what we would regard as a normal lifespan (Montagu, 1994; Batrinos, 2008). For example, Socrates before his untimely demise was in his 70s. Physicians had been directed to concoct potions to extend the life of emperors and the wealthy. In the Qin Dynasty, the emperor sent 500 young men and 500 young women to find the elixir of life in the legendary Penglai, the miraculous place of the immortals. Detailed descriptions of medicines for immortality were written in the book “Essential Formulas of Danjing Classics”. Some of these concoctions we would regard as remarkably toxic as they contain mercury or arsenic. Interestingly, this quest for longevity continues unabated and has now become a central pillar for modern health care. However, the perils persist with the unverified claims of a broad range of supplements or the off-target effects of therapeutics which maybe toxic or otherwise decrease longevity. Clearly, then as now an understanding of the fundamental biology and chemistry of aging is an essential goal for modern scientific research.

Despite the long history of the fascination of a long life, aging research as a systematic scientific effort is a recent affair. In the United States, the Aging Related Unit in the National Institutes of Health was formed in the 1940s, first in the NIH Division of chemotherapy, then moved to Baltimore City Hospital under the direction of Nathan Shock. In 1974 the National Institute of Aging (NIA) became an independent institute with a focus on aging biology and age related diseases. PubMed documents publications on aging as early as in 1925. In 1988, the first genetic locus *age-1* that modulates lifespan was identified in *C. elegans* (Friedman and Johnson, 1988), and 8 years later cloned and found to encode a PI3 kinase (Morris et al., 1996). Now there are a total of ∼487,000 articles using the search term “Aging” in PubMed, with ∼20,000 articles since 2020.

There has also been a long-standing interest associating aging with metabolism. Searching PubMed with “Aging and Metabolism” results in ∼188,685 articles, with 3,219 since 2020. Dietary restriction has been shown to affect longevity and age related illnesses in several organisms and model systems, with the effects on longevity dependent on genetic background (Mair et al., 2003; Liao et al., 2010; Cava and Fontana, 2013). At the molecular level, extended lifespan has been associated with insulin and IGF-1 receptor function, as well as *age-1*/PI3 kinase activity (Kenyon et al., 1993; Kimura et al., 1997). Modulation of sirtuins, which are NAD+ (Nicotinamide adenine dinucleotide) dependent enzymes, was reported to extend lifespan in yeast (Kennedy et al., 1995; Kaeberlein et al., 1999). AMPK (AMP activated protein kinase), a key sensor of metabolism and cellular energy, is required for lifespan extension in *C. elegans* in response to dietary restriction (Greer et al., 2007). Targeting the nutrient sensing pathway, the mTOR (mechanistic target of rapamycin) signaling pathway, using the inhibitor rapamycin, has been used to enhance longevity in several organisms, and shows efficacy when administered to aged mice (Harrison et al., 2009; Miller et al., 2011; Papadopoli et al., 2019). These studies suggest that the aging can be modified by changes in lifestyle or pharmacological intervention.

Observations that a deficit of mitochondrial function may result in energy shortage and accumulation of reactive species that are damaging to cellular structure and function inspired the “Mitochondrial dysfunction theory of aging” (Lemasters, 2005; Payne and Chinnery, 2015; Kauppila et al., 2017). Paradoxically, there are also observations that inhibition of mitochondrial respiration can extend lifespan. Reconciliation of these observations leads to the concept that the plasticity of metabolic pathways, which has a preprogrammed genetic component, is central in adapting to the environment and in turn impacts longevity (Kayser et al., 2004; Lapointe and Hekimi, 2008; Copeland et al., 2009; Yang and Hekimi, 2010). This is an idea captured in the “Hormesis theory of aging” (Ristow and Schmeisser, 2014; Yun and Finkel, 2014). Clearly, the term “mitochondrial dysfunction” is inadequate to describe the complexity of the adaptive capacity of the age regulated metabolic pathways. Additionally, a judicious inhibition of mitochondrial respiration may be required for activation of survival pathways (Chouchani et al., 2013). A better understanding of the role of metabolism in aging calls for more insights into the specifics of regulation of bioenergetics and metabolism which is now becoming feasible with the advent of sensitive and high precision technologies in these areas of research (Hill et al., 2019).

Although that “the Free Radical Theory of Aging” was proposed in 1956 based on the idea that free radicals can attack cellular constituents and thus may be a direct cause for aging (Harman, 1956), research linking aging to redox modulation is still evolving. With the realization that “free radicals” have a signaling role, it is clear that this basic hypothesis needs refinement to encompass new advances in redox biology and the recognition that all “free radicals” are not the same. Searching “Aging and Free Radical” has total of 13,754 articles, “Aging and oxidative stress” 19,044, “Aging and Redox” 9,139 articles. Many studies have challenged the idea that cellular oxidative damage due to “free radicals” or oxidative stress is a cause of aging. First, reactive oxygen species (or ROS) may be important in modulating aging, but the hypothesis lacks precision since it fails to identify “which species” contribute to aging or the mechanisms involved. This point is sometimes over-looked but similarly if we say “Genes” and “Proteins” are important in modulating aging, most of us will ask which gene(s) and which protein(s). Fortunately, technical advances are over-coming these barriers and allow specific hypotheses to be tested. Over the last 10 years the genetic regulation of redox related networks has also turned out to be remarkably complex. For example, one of the key regulators of redox modulatory proteins is Nrf2, which is a transcription factor that regulates genes encoding a subset of redox regulatory proteins, and is also a downstream target of insulin receptor and involved in lifespan regulation in *C. elegans* (Tullet et al., 2008). What is not predicted from the “Free radical theory of aging” is that increased expression of Nrf2 and its target antioxidant enzymes is detrimental for health and disease (Rajasekaran et al., 2011; Levonen et al., 2014; Dodson et al., 2015; Schmidlin et al., 2019). This key finding indicates that both “oxidative” and “reductive” stress, depending on their specificity, levels and cellular context, may have contrasting effects on aging-dependent processes.

In many aging related phenomena, including cellular senescence and perturbation circadian control, inadequacy in the autophagy and mitophagy pathways, also have strong connection to redox and metabolic regulation (Lopez-Otin et al., 2013). Autophagy is an intracellular degradation process that is highly regulated by a variety of signals including availability of metabolic substrates, cellular and the environmental redox landscape (Zhang, 2015; Klionsky et al., 2016). It is now clear that autophagy is a pathway that may remove and reverse cellular damage caused by oxidative stress and as such it is important to understand whether it is sufficiently active at the right place and at the right time (Lee et al., 2012; Giordano et al., 2014). Because of its central importance in health, disease and aging, the specific autophagic degradation of the mitochondria was identified as a specific process known as mitophagy (Lemasters, 2005; Redmann et al., 2014; Ma et al., 2020). Autophagy and mitophagy then play a key role in the quality control and turnover of lipids, proteins, and organelles, and their regulation modulates the metabolic and redox landscape (Dodson et al., 2013; Redmann et al., 2016). In aging tissues and age related diseases, these processes are unable to clear excess or dysfunctional proteins and organelles (Wong et al., 2020). Damaged organelles including mitochondria together with the accumulation of toxic proteins may further propagate cellular damage and contribute to the progression of age related diseases (Chen et al., 2020).

Aging research has been gaining momentum as better tools are developed and systems biology approaches are adopted. CRISPR/Cas techniques provide enhanced means of determining experimentally the functional consequence of gene disruption or mutation (Ran et al., 2013; Charpentier et al., 2019). These approaches can give insights into the networks that sense environmental signals that change cellular functions and thereby contributing to healthy aging or age related pathologies. Genomics, transcriptomics, proteomics, and metabolomics, some even at the single cell level, will aid in the understanding of the aging process in complex organs including the brain and the immune system (Aon et al., 2020; Zhang et al., 2020). High throughput bioenergetics analyses are now available which use small quantities of materials and even frozen samples and thus greatly extend the current studies of metabolism and its connection to aging (Dranka et al., 2011; Hill et al., 2012; Chacko et al., 2014; Redmann et al., 2018; Acin-Perez et al., 2020). For example, recent studies substantiated the connection of mitochondrial function, metabolism inflammation, and aging, and revealed the potential for a better understanding of this integrated regulatory network in attenuating age related diseases and promoting healthy aging (Bernard et al., 2018; Dunham-Snary et al., 2018; Rangarajan et al., 2018). In addition, a better understanding of the fundamentals of redox biology, and the improvement of techniques detecting and scavenging different reactive species are resulting in a rapid evolution of redox biology research (Kalyanaraman et al., 2012; Kalyanaraman, 2013; Afonso and Spickett, 2019). Advanced informatics methods have been developed, including GWAS, NetWAS, the transcriptome-metabolome-wide association study (TMWAS) and xMWAS platforms. These methods facilitate data integration, network visualization, clustering and differential network analyses of data from two or more omics dataset of genetic phenotypic, biochemical, or cell biological assays, which can reveal the whole organism changes that underlie the biology of aging (Beekman et al., 2013; Go et al., 2018; Uppal et al., 2018; Chacko et al., 2019; Roussarie et al., 2020; Smith et al., 2020). Integration of metabolism, redox biology with aging phenotypes will likely reveal novel nodes of regulation which can then identify new targets for healthspan extension interventions.

It is important to recognize that aging is not a single organ disease, and is highly dependent on the fact that tissues functionally interact and cross modulate. The blood and lymph circulate through the body and transport hormones, nutrients, cytokines, myokines, cell-free mitochondrial DNA, and other cellular metabolic products to other parts of the body (Barron and Pike, 2012; Coelho et al., 2019; Cunnane et al., 2020; Iske et al., 2020). Aging associated accumulation of the propionate metabolism product methylmalonic acid in the serum, may reprogram cancer cells to become more aggressive (Gomes et al., 2020). The microbiome contributes to a large portion of the total DNA/RNA in mammals and controls metabolism through its interaction with the diet and other environmental factors (Bernard et al., 2018; Bana and Cabreiro, 2019; Buford, 2020). Bacterial and viral infection, also alters the biology of the body and impact aging and age related diseases (Szaniawski and Spivak, 2020). Thus understanding the crosstalk between the gut, brain, liver, heart and muscle via the circulation is of critical importance to the understanding of aging biology (Lehallier et al., 2019). Since we cannot view age related pathologies only in isolated cells or tissue, we can also learn by observing nature. The existence of long-lived and short-lived species, for example, the naked mole-rat, different varieties of fish, clams, turtles, rodents, and centenarian humans, surely hold important clues to understanding how longevity and healthy living have been achieved (Austad, 2018).

An important contemporary research goal is to convert what we understand about metabolism and redox regulation in the context of aging into approaches to promote healthy aging. This approach can be surprisingly straight forward. For example, it has been shown that supplementation of mitochondrial TCA cycle metabolites, malate, fumarate, alpha-ketoglutarate, and oxaloacetate, supplementation of NAD+ precursor nicotinamide riboside extended lifespan in *Drosophila* and *C. elegans* (Belenky et al., 2007; Williams et al., 2009; Edwards et al., 2013; Chin et al., 2014; Zhang et al., 2016). Supplementation of alpha-ketoglutarate also decreases inflammation and frailty in mice, even when started at 18 months of age (Asadi Shahmirzadi et al., 2020). Not surprisingly, not all TCA cycle metabolites have the same effects. For example, accumulation of succinate is detrimental in the context of ischemia-reperfusion injuries (Chouchani et al., 2014). Dietary restriction, exercise, and circadian regulation have all been explored both in terms of metabolic mechanisms and with regard to reactive species. The networks involved in modifying lifespan are complex, and may be dependent on genetic, environmental, age, and other as yet unknown factors (Longo and Panda, 2016; Radak et al., 2019; Kepp et al., 2020; Perez-Matos & Mair, 2020). Pharmacological reagents that target autophagy and mitophagy can be tested and optimized against age related diseases and promote healthy aging (Galluzzi et al., 2017; Piskovatska et al., 2019). Compounds targeting the mitochondria, for example, MitoQ, and SS-31 have been explored for their potential for healthspan enhancement (Tate et al., 2019; Young & Franklin, 2019; Whitson et al., 2020). Senolytics have been shown to attenuate cell-free mitochondrial DNA release, which then decreases the detrimental immune responses associated with aging (Iske et al., 2020). Mitochondria not only can be targeted to improve metabolism and healthspan, but also can generate the mitochondrial-derived peptides (MDPs) that can regulate metabolism and health (Reynolds et al., 2020).

Advancement of hygiene, food and environmental safety, health care, and a better understanding of aging biology and aging interventions are at such a stage that it has been suggest that the individuals who will reach the age of 150 are already living (https://www.stevenaustad.com/). It is still not clear why age is the single most important risk factor for many diseases including but not limited to: cancer, cardiovascular and neurodegenerative diseases. This is a new exciting era in which enabling technologies can provide exciting new insights into the mechanisms and biology of aging. It is likely that research linking aging to metabolism and redox regulation will feature prominently in the next decade. The grand challenge is then to understand the networks linking metabolism, and redox biology to cell aging and organismal aging and to target specific metabolic and redox networks for the promotion of a healthy lifespan.

## Author Contributions

The author confirms being the sole contributor of this work and has approved it for publication.

## Conflict of Interest

The authors declare that the research was conducted in the absence of any commercial or financial relationships that could be construed as a potential conflict of interest.
